# A first look at multiple institutional affiliations: a study of authors in Germany, Japan and the UK

**DOI:** 10.1007/s11192-017-2257-6

**Published:** 2017-01-28

**Authors:** Hanna Hottenrott, Cornelia Lawson

**Affiliations:** 1 TUM School of Management, Technische Universität München, Arcisstraße 21, 80333 Munich, Germany; 2 Centre for European Economic Research (ZEW), Mannheim, Germany; 3 Department of Managerial Economics, Strategy and Innovation, K.U. Leuven, Leuven, Belgium; 4 School of Management, University of Bath, Quarry Rd, Bath, BA2 7AY UK; 5 BRICK, Collegio Carlo Alberto, Moncalieri (TO), Italy

**Keywords:** SCI, Multiple affiliations, Dual appointment, International collaboration, Science-industry collaboration, High-impact research, I23, J45, O30

## Abstract

This study sheds light on the unexplored phenomenon of multiple institutional affiliations using scientific publications. Institutional affiliations are important in the organisation and governance of science. Multiple affiliations may alter the traditional framework of academic employment and careers and may require a reappraisal of institutional assessment based on research outcomes of affiliated staff. Results for authors in three major science and technology nations (Germany, Japan and the UK) and in three fields (biology, chemistry, and engineering) show that multiple affiliations have at least doubled over the past few years. The analysis proposes three major types of multiple affiliations that depend on the structure of the research sector and its international openness. Highly internationalised and higher education-centred affiliations are most common for researchers in the UK whereas Germany and Japan have stronger cross-sector affiliation patterns. International multiple affiliations are, however, still more common in Germany compared to Japan which is characterised by a domestic, cross-sector affiliation distribution. Moreover, multiple affiliation authors are more often found on high impact papers, particularly in the case of authors from Japan and Germany in the fields of biology and chemistry.

## Introduction

Recent literature has emphasised the importance of intra- as well as inter-institutional collaborations for academic research, which has been documented by the increase in team sizes and cross-institutional collaborations on co-authored papers (Adams et al. 2005; Wuchty et al. 2007; Jones et al. 2008). In this academic environment that promotes collaboration and mobility (Fernandez-Zubieta et al. 2016), multiple affiliations are increasingly recognised as facilitating knowledge exchange (ESF 2013) and have been said to become more widespread (Enders and Musselin 2008). Nonetheless, to date there has been little systematic assessment of the extent and patterns of these multiple affiliations, primarily due to the lack of sufficient bibliometric data which would allow for determining such links (Katz and Martin 1997).

There are several factors that may contribute to multiple affiliations. Individual scientists may seek affiliations to gain access to additional research resources or networks, as affiliation to an institution is closely linked to resource access, research infrastructure and career opportunities (Long 1978; Long and McGuinnis 1981; Fox 1983; Stephan 2012), but may also be motivated by personal financial benefits (Stephan 2012). While co-authorship can be an effective way of expanding a research group’s competencies, co-affiliation may constitute a way of forming stronger ties between researchers and institutions (ESF 2013). Lander (2015) for instance, found some evidence that in the case of infection and immunity researchers in Canada multiple affiliations facilitate co-authorship. Co-affiliation may also reduce the (administrative) burden of using an institution’s research infrastructure compared to collaboration through co-authorship alone. Furthermore, researchers may seek additional affiliations to increase the visibility and geographic reach of their work, especially if they are placed in more peripheral regions or institutions.

On the institutional side, performance assessments have led universities across the globe to become more proactive in attracting the most prolific researchers in a bid to enhance their position in national and international rankings (Stephan 2012). Offering the option of additional affiliations to the most able researchers in a field may provide institutions with access to ‘frontline’ researchers (ESF 2013). For example, universities in China and Saudi Arabia created special part-time positions to attract leading foreign scholars to enhance their prestige and ranking, which saw an increase in papers from both countries, but with science that did not always originate there (Xin and Normile 2006; Bhattacharjee 2011). Affiliations are also offered by past employers in an effort to maintain links with alumni employees and may therefore constitute a direct consequence of increased mobility. Public, non-profit and private sector research centres moreover offer affiliations in order to foster mobility and facilitate research collaborations and knowledge exchange with the academic sector (ESF 2013).

The drivers (such as resource access, personal finances or institutional competition) and implications (such as collaboration or research advancement) of multiple affiliations likely differ by the type of affiliation under consideration, as well as by the institutional contexts in which these affiliations form. Thus, for any assessment of their potential implications, one first requires a better understanding of the forms that multiple affiliations take and how they differ between disciplines and countries with distinct science governance structures.

Addressing the current lack of evidence regarding multiple affiliations, this study proposes an analysis of institutional affiliations based on scientific publications that makes use of a new author-institution tag available in Web of Science (WoS) since 2008. The new tag allows us to unambiguously link authors to their institutions and to determine whether an author has multiple affiliations.^1^ We then analyse the types of multiple affiliations in terms of cross-sector and cross-country affiliations. Further, incorporating citation data we investigate the link between multiple affiliations and publication impact. Looking at authors in Germany, Japan and the UK (thus straddling three major science and technology nations) and in three fields (biology, chemistry, and engineering), our results demonstrate that multiple affiliations have increased over the past few years. Yet, the types of multiple affiliations depend on the structure of the research sector and its international openness.

While this analysis can only provide a first look at the extent and structure of multiple affiliations, we anticipate the findings will encourage more research into their contractual and organisational nature. We also hope to spur discussion regarding academic employment and affiliations that no longer require the day-to-day commitment of academics, with implications for institutional assessment (currently based on research outcomes of affiliated staff) and the funding of academic research, both nationally and internationally.

## Data and methods

The data used in this study are author-institution pairs retrieved from Web of Science. First we select a set of journals based on the 2013 journal citation report (JCR, Thomson Reuters) in three fields: bioscience, chemistry, and engineering. Journals listed in the JCR are sorted by *eigenfactor* score, a rating of journal importance based on the number of incoming, journal-impact-weighted citations, that enables us to consider journals across all quality spectra. The bottom 50% of the *eigenfactor* distribution is discarded as they represent primarily national journals of little importance within the field. The remaining journals are split into four quartiles of the *eigenfactor* distribution for each scientific field and five journals are randomly drawn from each quartile, thus obtaining three samples of 20 journals by field, stratified by *eigenfactor* score. For each journal we check on a subsample of articles whether addresses are correctly listed and we drop and replace journals with any address inconsistencies. Documents published in each of the selected journals and with an address in one of the three selected countries (Germany, Japan and the UK) are downloaded from WoS for the years 2008–2014. The retrieval relies on author addresses as listed in WoS. Overall, we consider records of 13,827 bioscience articles, 12,155 chemistry articles and 5274 engineering articles.

Each author on the selected publications is attributed one or more institution based on the [C1] author address field in WoS. The [C1] field provides address information separately for each author and lists more than one address per author where this occurs. We split each publication record by author and address to build a table with author-institution lines. These author-institution records are checked for any address inconsistencies, i.e. whether address information is missing for one or more authors. Publications that do not report addresses for all their authors are excluded. The number of excluded publications is 1351, or 3.8% of all publications.

Only authors with at least one address in Germany, Japan or the UK are retained. In total we identify 36,035 authors with an address in Germany, 57,604 with an address in Japan, and 31,648 with an address in the UK. It is important to note that this study does not attempt to disambiguate author names and the same author can appear in the data multiple times. As the goal is to provide an application of the [C1] field for measuring multiple affiliations and to gain first insights and identify general trends, no author disambiguation was performed. However, results are robust to the deletion of all repeated names within one subject area and year, or to the deletion of all duplicate names in general.

All addresses appearing in the data are coded by institution type and country. In doing so, each institution is assigned a unique code to ensure that multiple addresses are truly different and to exclude multiple affiliations ‘within’ the same institution. The coding was undertaken semi-manually, meaning that a search algorithm first identified address entries containing word elements such as “UNIV” and marked these as universities. All entries were then checked manually and organisation names searched online to assign institution types.^2^ We not only coded institutions in the three countries of interest but also institutions in other countries if an author had a secondary affiliation there. In total we identified 4064 different institutions. These institutions were coded as belonging to the higher education sector (HEI),^3^ public or semi-governmental research institutes (PRO), non-profit research institutes (NGO), private sector institutions, government, or other institutions (such as industry associations).^4^ Each institution was moreover assigned a country code. The maximum number of affiliations at truly different institutions observed for one author is seven, while the maximum number of different institution types is three.

In addition, the number of citations received up to 31st March 2016 is collected for all articles in the sample to measure the impact of multiple affiliations. This means that we have different citation windows depending on the year of publication. As the citation count is highly field and year sensitive, we follow Lee et al. (2015) and consider papers that are in the top 1% of citations in their field in each year (as of March 2016) as papers with high scientific impact. This alternative measure corrects for any year or subject bias of the pure citation count. Only 3163 authors in our sample appear on articles that are in the top 1%, we therefore also consider the top 10% as an alternative measure (21,139 authors). This method of determining impact may be preferable to pure citation counts as it is less sensitive to year and field differences.

## Results

### Trends in multiple affiliations

Table 1 shows the total number of authors reported on the selected publications by country and field, as well as the number and proportion of authors that report more than one institutional address. Of the more than 118,000 authors in the sample, 7.2% have more than one institution attached, with some differences across countries and subject areas.^5^ The proportion of authors with multiple institutional addresses is highest with more than 9% of authors in biology and chemistry in the case of Germany, and biology in the case of the UK. This already suggests some country and subject-specific differences regarding the extent of multiple affiliations.

**Table 1:** Number of authors by field, and number of authors with addresses in multiple institutions (based on author-publication pairs for the years 2008–2014)

Country	Subject	No. of authors	No. of authors with multiple affiliation	Proportion (%)
Japan	Biology	34,294	1993	5.81
	Chemistry	18,242	1297	7.11
	Engineering	4273	350	8.19
Germany	Biology	12,180	1183	9.71
	Chemistry	16,034	1480	9.23
	Engineering	5971	417	6.98
UK	Biology	10,317	1050	10.18
	Chemistry	11,069	630	5.69
	Engineering	6355	355	5.59
Total		118,532	8553	7.22

**Fig. 1:**
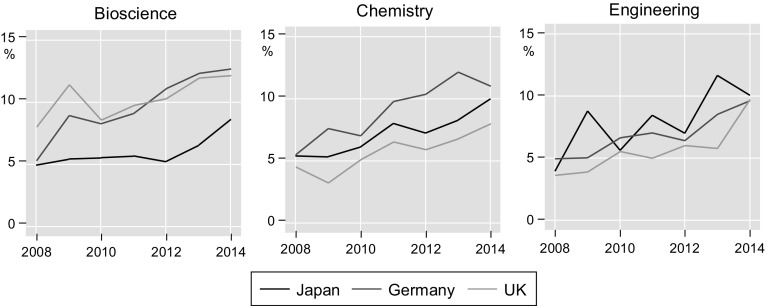
Share of authors with multiple affiliations, 2008–2014, by country and field. *Note* Colour scheme from Bischof (2016)

Author-institution pairings are available from 2008 onwards which means that we can also observe whether there is a trend towards more institutional affiliations since then. Figure 1 shows how the share of authors with more than one affiliation address evolved over the 2008–2014 period. We see an upward trend in multiple affiliations in all scientific fields and countries from about 5% in 2008 to 10% in 2014. For Japanese authors in bioscience, however, this increase only happened after 2012 and multiple affiliations are still lower than for bioscience authors in Germany and the UK.

These results of a significant and growing proportion of authors with multiple affiliations also corroborate the increasing importance of studying multiple affiliations in the context of scientific research.

### Main affiliations in the three countries

The research structure differs between the three countries and subject areas, and these differences are reflected in the types of institutions that publish academic research. Table 2 reports the different forms of institutional affiliations within the three countries for all 118,532 authors. For this purpose all multiple affiliations outside the focus country are excluded and only affiliations within each of the three countries considered. We observe, that most authors have their address within a HEI. This share is highest for authors in the UK, where 86% of authors have at least one address within a HEI.^6^ The share of authors with an address in a HEI is lowest in Germany (69%) and especially in engineering, where only 55% of authors are affiliated with a HEI. Outside the higher education sector authors are primarily affiliated to PROs. In Germany, PROs account for 22% of all author addresses, which has its root in a large non-academic public research sector in Germany. In Japan, PROs account for 12% and in the UK for just 3% of all authors. NGOs are an important affiliation only amongst bioscience authors in the UK, through institutions such as the Wellcome Trust. Here, they account for almost 8% of authors. Private sector authors are found mostly in engineering in Germany and Japan with more than 16% of authors, and in bioscience in Japan with 15%. In the UK they account for just 6% of authors. Government appears as a frequent affiliation amongst engineering and chemistry authors in Germany and the UK with 3–7% of authors.

**Table 2:** Author affiliations ‘within’ the three countries (in % of all authors)

Country	Discipline	HEI	PRO	NGO	Private	Government	Other
Japan	Bioscience	75.62	11.10	0.78	14.95	0.84	0.13
	Chemistry	82.43	13.01	1.13	6.34	0.89	0.13
	Engineering	73.06	13.37	0.82	16.57	0.54	0.21
Germany	Bioscience	72.50	24.80	0.27	4.98	1.35	0.21
	Chemistry	71.30	19.08	0.81	8.15	4.52	0.04
	Engineering	55.38	23.87	1.09	16.65	6.88	0.02
UK	Bioscience	81.73	5.68	7.57	4.37	2.01	0.10
	Chemistry	87.51	1.97	0.45	7.06	3.84	0.17
	Engineering	90.07	2.03	0.02	5.90	2.91	0.03

Row sums are larger than 100% as authors can belong to more than one institution type. Only addresses within the three countries are considered. Both, single and multiple affiliation authors are considered

The address breakdown by country and sector reflects the research activities of the various sectors in the three countries as well as the research structure. Higher publication participation amongst private sector authors in Japan and Germany compared to the UK suggests that more commonality between sectors and opportunities for joint research may exist there, which may also be conducive to multiple affiliations.

### Cross-sector and international affiliations

**Fig. 2:**
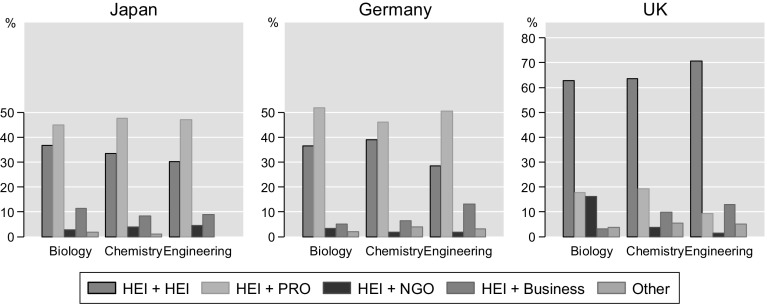
Cross-sector affiliations as share of authors with multiple affiliations, by country and field. *Note* Bar sums can be larger than 100% as authors can belong to more than two institution types. Colour scheme from Bischof (2016)

Multiple affiliations can occur across all sectors. Figure 2 reports the share of authors with two or more HEI affiliations, and HEI and selected cross-sector affiliations over all multiple affiliations by countries and fields, including affiliations with institutions abroad. Only the most common combinations are reported. Amongst UK authors, more than 60% with multiple affiliations have a cross-HEI affiliation, i.e. belong to two or more HEI, regardless of academic field. For authors with at least one address in Germany or Japan, this share is much lower at fewer than 40% of multiple afilliation authors. Instead, cross-sector affiliations, especially with PROs, are more often observed. This is reflective of the stronger public research sectors in the two countries, which also seems to extend into joint appointments. For UK authors, cross-affiliations with the non-governmental charity research sector (NGO) are of importance in the biosciences. The share of authors with joint HEI and private sector affiliations is highest in engineering (12%) and for authors in Japan (10%), that is, in those areas, where private sector publications are more often found. Multiple affiliations with other sectors, such as national and local government agencies are less often found. Also cross-affiliations that do not include at least one university are rarely observed. Only 5% of authors with multiple affiliations do not concern HEI authors.

Additional affiliations can not only be in a different sector but also with an institution abroad. We propose to plot affiliations along their international and cross-sector dimensions, to quantify the difference in cross-affiliations between countries and fields. We do this by plotting the share of multiple affiliation authors with a cross-sector affiliation on the *x*-axis and the share of multiple affiliation authors with an international affiliation on the *y*-axis for each country and field combination.

**Fig. 3:**
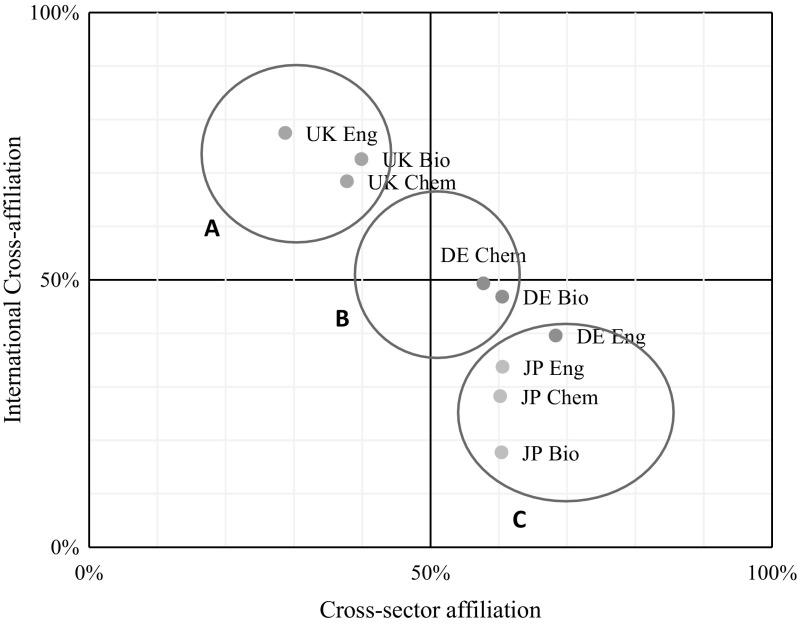
Cross-sector and international cross-affiliations as share of authors with multiple affiliations, by country and field

Figure 3 shows that cross-sector affiliations are more common amongst authors with at least one address in Japan or Germany, as already seen in Fig. 2. For authors with an address in Japan the domestic nature of multiple affiliations is particularly strong, while authors in Germany show slightly higher levels of internationalisation with 40–50% of multiple affiliations involving an institution abroad. Authors with at least one address in the UK, on the other hand, are characterised by little cross-sector but high international cross-affiliations. This means that authors in the UK primarily cross-affiliate with HEIs abroad. The graphical representation in Fig. 3 thus shows that in this three-country – three-field analysis we can observe three classes of multiple affiliations: (A) a highly internationalised, HEI-centred affiliation distribution as represented by authors in the UK, (B) a balanced affiliation distribution as seen in Germany, and (C) a domestic, cross-sector affiliation distribution as seen in Japan.

### Impact and multiple affiliations

Previous literature has linked collaboration to higher research quality and impact in terms of citations (Katz and Hicks 1997; Wuchty et al. 2007). Using publication level information regarding citations received up to 31st March 2016, we can investigate whether multiple affiliations too are associated with higher impact publications. One may expect a correlation between author numbers and multiple affiliations, and co-author numbers thus to act as confounding factor, however, there is no significant correlation between the two (6.51 vs. 6.50 authors, *p* > 0.1), with the exception of chemistry in Japan and Germany.

Table 3 reports the differences in the number of citations and the share of top-impact papers between those with and without multiple affiliations. It shows that, in the case of Japan, authors with multiple affiliations have higher citation numbers and are more often found on top impact publications in bioscience and chemistry. In the case of Germany, only bioscience publications with multiple affiliation authors have statistically significantly more citations or top impact articles than those with single affiliation status. Finally, UK authors with multiple affiliations do not receive more citations or have more top 1% articles. They do, however, still publish more articles in the top 10% in bioscience and engineering, but not chemistry. These correlations suggest that for UK authors their primarily international affiliations are less associated with higher article impact. Bioscience and chemistry authors in Japan, on the other hand, show higher publication impact that may be due to their links with PROs, but may also represent funding relationships.

**Table 3:** Citation impact of authors with single versus multiple affiliations

Discipline	Country	Citation numbers			Top 1%-cited (in %)			Top 10%-cited (in %)		
		Single-affil	Multi-affil	*t* test	Single-affil	Multi-affil	*χ* ^2^ test	Single-affil	Multi-affil	*χ* ^2^ test
Japan	Bioscience	9.5	18.3	***	0.8	4.3	***	4.8	14.6	***
	Chemistry	12.1	18.4	***	0.7	1.2	**	11.7	24.6	***
	Engineering	6.0	6.7		0.5	2.0	***	9.7	11.4	
Germany	Bioscience	26.0	34.8	***	5.9	11.9	***	25.5	38.5	***
	Chemistry	19.5	21.6		2.3	2.8		23.1	31.9	***
	Engineering	6.9	6.7		0.3	0.0		12.1	13.4	
UK	Bioscience	35.5	33.0		10.0	9.9		35.5	40.7	***
	Chemistry	20.9	17.5	**	2.2	1.1	*	25.6	27.0	
	Engineering	8.0	8.3		1.2	2.0		14.2	19.7	***

*** (**, *) indicate significance levels of 1% (5%, 10%) using a *t*-test or a *χ* ^2^ test. Significance levels remain in ANOVAs that control for author counts

## Conclusion

This paper studied multiple affiliations of authors in research publications. Results for three scientific fields (biology, chemistry and engineering) and three countries (Germany, Japan and the UK) showed that multiple affiliations are widespread and have increased in all fields and countries during the period 2008–2014.

We found that multiple affiliations reflect the dynamics of the research sector in specific countries and proposed a classification of the cross-sector and international dimension of author affiliations. To summarise, we find three types of multiple affiliations that can be classified as (A) a highly internationalised, HEI centred affiliation distribution as represented by researchers in the UK, (B) a balanced affiliation distribution as seen in Germany, and (C) a domestic, cross-sector affiliation distribution as seen in Japan. These results suggest that cross-sector affiliations are highest in countries and fields with a large non-university research sector, while cross-country affiliations are highest in countries with an international research base. An analysis of other countries may find additional types. However, the occurrence of low cross-sector affiliations paired with low internationalisation, that is, where academic authors are primarily affiliated with other domestic universities, may be limited by academic employment contracts which generally still limit such arrangements.

These observed differences have consequences for the types of networking that can be achieved through multiple affiliations in different countries. For example, international affiliations may help to preserve links to ‘frontline’ research institutions, while cross-sector affiliations may be more conducive to knowledge transfer and mobility between sectors (ESF 2013). Our results did, however, show that most multiple affiliations of academics are with other universities or with PROs, including in the cases of Japan and Germany. The role of multiple affiliations as a facilitator for knowledge transfer between distinct sectors (ESF 2013) may therefore be rather limited.

We also find some evidence that multiple affiliation authors are more often found on high impact papers. This complements existing findings of a positive correlation between collaboration and citations (Katz and Hicks 1997; Wuchty et al. 2007). Regardless of author team size, citation numbers are higher for authors with multiple affiliations in the biosciences and chemistry in Japan and Germany. In the UK, however, we only find smaller correlations. Resource access considerations may thus be the driver for multiple affiliations in Germany and Japan, while in the UK this may not be the case as they may simply have less to gain from additional affiliation due to their already higher citation numbers.

Nevertheless, several limitations apply. For example, this analysis does not determine whether the association between citations and affiliations found in Japan and Germany is driven by the affiliation itself, through access to human or other resources, or whether perhaps more able academics are more likely to engage in additional affiliations. To investigate these causal relationships, more information on individual authors is required. Moreover, it is not clear how well publication data can capture multiple affiliations. While institutions may encourage researchers to list them, this may be dependent on the rewards offered. Authors may also take strategic decisions regarding the affiliations they list, depending on the journal or the type of research undertaken. A longitudinal study of authors is therefore encouraged to uncover authors’ choices regarding institution address selection. It is further not clear from publication data what the nature of multiple affiliations is, i.e. whether they are due to second appointments, visiting periods, research funding, or other types of arrangements. We also cannot be certain about an author’s home institution when multiple addresses are listed. This may affect the interpretation of some of the findings as foreign university researchers could be affiliating with PROs, private sector institutions or others in one of the three countries in this analysis. Publications may also not represent the preferred dissemination method for academic authors that are also affiliated with the private or government sector. These may rely on alternative dissemination channels such as reports and thus not be captured through publications in top field journals.

Regardless, our results document multiple affiliations as another factor in the international and inter-institutional collaboration of academic research as well as its correlation with high impact research. These insights thus contribute to the discussion about increases in team sizes and cross-institutional collaborations on co-authored papers (Adams et al. 2005; Wuchty et al. 2007; Jones et al. 2008).

The findings also have direct implications for research policy. On the one hand there are multiple affiliations that work as a reallocation mechanism between institutions, which may benefit resource use efficiency, knowledge exchange and collaboration, and should therefore be encouraged. On the other hand there are assessment- or income-driven affiliations, which could give rise to institutions that act purely as a PO-Box for researchers with little or no actual research undertaken there. Such affiliations can distort institutional performance measures and rankings and have ethical implications, due to a lack of research contribution from the additional institution (Safaei et al. 2016). Multiple affiliations may also reflect (or may be a symptom of) a decline of institutional support for academics, especially regarding resource constraints in university-based research or the casualisation of the academic profession, which require academics to seek resources and work roles outside their main institution. We therefore encourage more research into multiple affiliations in other country and field contexts, and are confident that our analysis will encourage a discourse regarding their scientific and institutional impact.

## Footnotes

1 This tag has previously been used by Lander (2015) to determine multiple affiliations amongst infection and immunity researchers in Vancouver, Canada.

2 Coding of institution types in Japan additionally made use of WoS address dictionaries provided by the National Institute of Science and Technology Policy (NISTEP) (http://www.nistep.go.jp).

3 In the case of Japan this denotes national, regional (public) and private universities; in Germany it includes universities, technical universities and universities of applied sciences (UAS).

4 Hospitals were assigned to one of the institution types depending on their organisational structure. The majority were university teaching hospitals and thus assigned to HEI.

5 The shares are higher at 12% if we consider multiple affiliations within the same institution. However, such intra-institutional multiple affiliations are not the focus of this analysis.

6 This share is higher than previous research by Katz and Hicks (1996) suggested. The difference may be due to changes in the UK public research sector organisation, where many previously independent research council laboratories and hospitals have experienced a closer integration with universities.
